# Short-Term Changes in Lower Extremity Volume After Radiofrequency Diathermy in Women with Idiopathic Lower Extremity Edema: A Randomized Crossover Trial

**DOI:** 10.3390/jcm15207974

**Published:** 2026-10-15

**Authors:** Ji Soo Baik, Cherrie Ahn, Sung Hwa Ko, Jung-Lim Lee, Ji Hong Min, Yong-Il Shin

**Affiliations:** 1 Biomedical Research Institute, Pusan National University Yangsan Hospital, Yangsan 50612, Gyeongsangnam-do, Republic of Korea; zisoo@pusan.ac.kr (J.S.B.); ahn8380@pusan.ac.kr (C.A.); ijsh6679@gmail.com (S.H.K.); lim6668@naver.com (J.-L.L.); 2 Department of Rehabilitation Medicine, Pusan National University Yangsan Hospital, Yangsan 50612, Gyeongsangnam-do, Republic of Korea; 3 Department of Rehabilitation Medicine, Pusan National University School of Medicine, Yangsan 50612, Gyeongsangnam-do, Republic of Korea; 4 Department of Convergence Medical Science, Pusan National University School of Medicine, Yangsan 50612, Gyeongsangnam-do, Republic of Korea

**Keywords:** thermotherapy, skin temperature, thermal stimulation, physiological response, noninvasive therapy

## Abstract

**Background**: Idiopathic lower extremity edema, characterized by recurrent swelling without an identifiable underlying disease or medication-related cause, is associated with altered fluid distribution and peripheral circulation. Radiofrequency diathermy (RFD) may influence local thermal and circulatory conditions; however, its short-term effects on edema-related parameters remain unclear. This study aimed to evaluate the short-term changes in lower extremity volume and related physiological measures following RFD in women with idiopathic lower extremity edema. **Methods**: A randomized, assessor-blinded, sham-controlled crossover study was conducted in 14 women with idiopathic lower extremity edema. Participants received both active RFD and sham treatments once daily for three consecutive sessions, with the two treatment periods separated by a 4-week washout period. Leg volume, circumference, body weight, pain intensity, and skin temperature were measured before and after each session. **Results**: Compared with the sham condition, the active condition was associated with greater reductions in right leg volume (active: −104.0 mL; sham: −14.5 mL; *p* = 0.013) and left leg volume (active: −81.1 mL; sham: +17.4 mL; *p* < 0.001). Compared with sham, the active condition also showed greater reductions in pain intensity, body weight, and right calf circumference (all adjusted *p* < 0.05) and greater skin temperature increases in all regions except the bilateral anterior thighs (all *p* < 0.05). **Conclusions**: In women with idiopathic lower extremity edema, the active condition was associated with greater short-term reductions in bilateral leg volume than the sham condition, accompanied by changes in skin temperature and related physiological measures.

## 1. Introduction

Idiopathic lower extremity edema is a common clinical condition characterized by swelling and discomfort in the absence of an identifiable underlying disease or medication-related cause, typically presenting as persistent or recurrent edema rather than transient swelling [1,2]. Although it is often considered a mild condition, it can cause substantial daily discomfort and functional limitations and is more commonly observed in women. Idiopathic edema is also associated with marked fluctuations in swelling and body weight over the course of the day, with its pathophysiology thought to involve complex interactions among fluid distribution, capillary permeability, and peripheral circulatory regulation [3,4,5].

Idiopathic lower extremity edema is generally considered a diagnosis of exclusion and should be distinguished from edema caused by chronic venous insufficiency, cardiovascular disorders, hepatic dysfunction, renal disease, or medication-related factors [6]. Therefore, even in the absence of identifiable structural lesions or systemic disease, patients may experience recurrent edema accompanied by symptoms such as heaviness, tightness, pressure, or pain [7], features that distinguish the condition from acute inflammatory edema [5,8].

Unlike acute inflammatory edema, which is driven primarily by tissue injury, inflammatory mediator activation, and increased capillary leakage [8], idiopathic edema is more closely associated with disturbances in fluid regulation and distribution, impaired peripheral circulatory control, and changes in capillary permeability [5]. Interventions that modulate fluid redistribution and the local circulatory environment may therefore be clinically relevant for idiopathic lower extremity edema, alongside approaches that limit further fluid accumulation [1,5].

Current management strategies for idiopathic lower extremity edema are largely supportive and include leg elevation, sodium restriction, compression therapy, and massage [1,7,9,10,11,12]. These approaches aim to alleviate symptoms by facilitating fluid movement and improving peripheral circulation [1,7,9,10,11,12]. However, their clinical effectiveness may vary with implementation, adherence, and treatment duration [9,10,11,12]. Moreover, because these approaches mainly focus on relieving symptoms or preventing further fluid accumulation, evidence for interventions that directly target fluid distribution and peripheral circulatory regulation, which may be more central to the pathophysiology of idiopathic edema, remains limited [8].

Unlike superficial heating modalities, radiofrequency diathermy (RFD) can generate heat in deeper soft tissue layers, not only at the skin surface [13,14]. Previous studies of 448 kHz capacitive–resistive monopolar radiofrequency have reported physiological responses at depths of approximately 2–3 cm, supporting its effects beyond the superficial skin layer [15]. It has been used in physical therapy and rehabilitation for pain management, tissue recovery, and improvement in joint mobility [16,17,18,19,20,21,22,23,24]. The physical effects of RFD arise from the interaction of high-frequency electromagnetic energy with ions and polar molecules within tissues, producing resistive energy losses that are converted into heat [24,25]. Unlike superficial heat modalities, RFD can generate endogenous heat within deeper tissue layers, and the resulting thermal responses may increase blood perfusion, modulate vascular reactivity and microcirculation, enhance lymphatic drainage and venous return, ultimately contributing to fluid redistribution within treated tissues [25,26,27]. This may be particularly relevant in idiopathic lower extremity edema, in which functional dysregulation of fluid homeostasis plays an important role.

Compared with conventional conservative interventions, RFD is distinguished by its ability to induce thermal responses within deeper tissue layers through the application of electromagnetic energy, thereby potentially influencing the local circulatory environment and interstitial fluid dynamics [24,25]. Previous studies of noninvasive radiofrequency-based therapies have reported beneficial effects in edema-related conditions, including reductions in limb circumference or swelling and improvements in tissue temperature and local perfusion [28,29,30,31]. However, most of these studies have focused on lymphedema or lipedema, which differ from idiopathic lower extremity edema in pathophysiology and clinical characteristics. Treatment protocols and outcome measures have also varied considerably across studies [28,29]. Therefore, it remains unclear whether RFD produces comparable short-term changes in lower extremity volume and edema-related symptoms in patients with idiopathic lower extremity edema. This limited evidence warrants preliminary investigation under controlled conditions. The present study was designed to address this gap by evaluating the short-term effects of RFD on lower extremity volume, circumference, skin temperature, body weight, and edema-related pain using a randomized, sham-controlled crossover design.

Clinically meaningful thresholds for changes in lower extremity volume or circumference are not consistently defined across edema-related conditions [32,33]. Numerical changes in limb size alone may therefore not fully capture clinical benefit in patients with idiopathic lower extremity edema. Because these patients commonly experience recurrent leg heaviness, aching, pressure, tightness, and edema-related pain, improvement in these symptoms may also represent a clinically meaningful treatment effect [1,2,4,7]. Thus, the clinical relevance of treatment in idiopathic lower extremity edema should be interpreted in terms of both objective changes in limb volume or circumference and patient-reported symptom improvement.

We hypothesized that RFD might alter local temperature and blood flow in patients with idiopathic lower extremity edema, thereby reducing tissue tension, alleviating fluid retention, reducing edema, and relieving pain intensity. Accordingly, this study aimed to evaluate short-term changes in lower extremity volume, related physiological measures, and pain intensity following RFD in women with idiopathic lower extremity edema.

## 2. Materials and Methods

### 2.1. Study Design and Ethics

**Figure 1:**
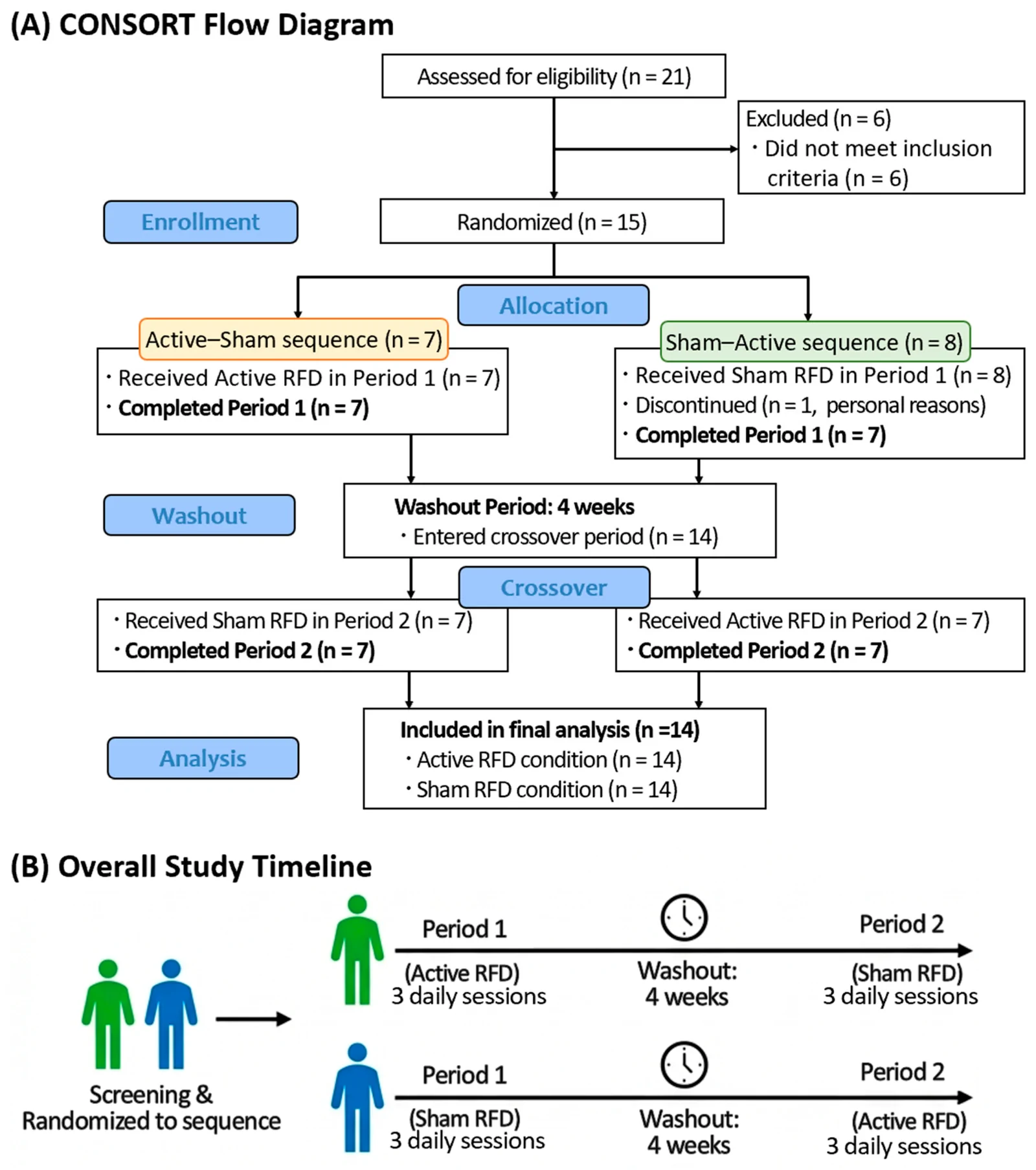
Study flow and crossover trial design. (**A**) CONSORT flow diagram; (**B**) randomized crossover trial protocol. Pre- and postintervention assessments were performed immediately before and after each intervention session. The intervention was administered once daily for 3 consecutive days. RFD, radiofrequency diathermy.

The present study was a prospective, randomized, assessor-blinded, sham-controlled crossover clinical trial. The first participant was enrolled on 21 September 2020, and the study was completed on 30 June 2021. The study flow diagram, prepared in accordance with the CONSORT guidelines, is presented in Figure 1. The protocol was approved by the Institutional Review Board of Pusan National University Yangsan Hospital (IRB No. 03-2020-001) and by the Ministry of Food and Drug Safety of the Republic of Korea (Approval No. 1025). The trial was registered at ClinicalTrials.gov (ID: NCT05185193). Participants were recruited through institutional advertisements and outpatient referrals at Pusan National University Yangsan Hospital. The study was conducted in accordance with the Declaration of Helsinki, and all participants provided written informed consent before enrollment.

### 2.2. Sample Size

Given the lack of prior research on noninvasive RFD for lower extremity edema, a conventional power-based sample size calculation was not feasible. Following recommendations for pilot studies, a minimum sample size of 12 participants was considered appropriate [34]. To allow for a potential dropout rate of approximately 20%, a target sample of 15 participants was established.

### 2.3. Inclusion Criteria and Exclusion Criteria

Participants diagnosed with idiopathic edema based on clinical evaluation and the McKendry criteria were recruited. The inclusion criteria were as follows: (1) idiopathic edema, defined as a McKendry score of ≥15 [35]; (2) treatment scheduled between 8 and 18 days after the onset of menstruation, such that menstruation was not expected during the treatment period. The exclusion criteria were as follows: (1) presence of a metallic pacemaker; (2) menstruation or possible pregnancy; (3) cardiovascular, hepatic, or renal disorders; (4) active bleeding or hematoma; (5) uncontrolled hypertension; (6) epilepsy or clinically relevant neurological conditions, such as vasovagal syncope/syndrome, Guillain–Barré syndrome, or peripheral neuropathies; and (7) use of weight-loss drugs, venoactive medications, or supplements that could affect edema or vascular function, including flavonoids or other phlebotonics.

### 2.4. Intervention

Participants were randomly assigned, using a computer-generated randomization schedule, to one of two treatment sequences: active–sham or sham–active. The allocation sequence was generated by an independent researcher who was not involved in participant recruitment, intervention administration, outcome assessment, or data analysis. Treatment assignments were concealed until each participant had been enrolled.

Participants received each condition (active or sham) once daily for three consecutive sessions (S1, S2, S3), with each session lasting approximately 30 min. To minimize potential carryover effects between treatment periods, a 4-week washout period was prespecified. This interval exceeded the washout periods used in previous crossover studies and was considered sufficient to minimize residual treatment effects [15,36,37]. It was also selected to reduce potential menstrual-cycle-related variability, with treatment sessions scheduled outside the expected menstruation period.

Participants were additionally instructed to avoid food and beverages other than water, vigorous physical activity, and caffeine before each measurement session to reduce short-term physiological variability.

**Figure 2:**
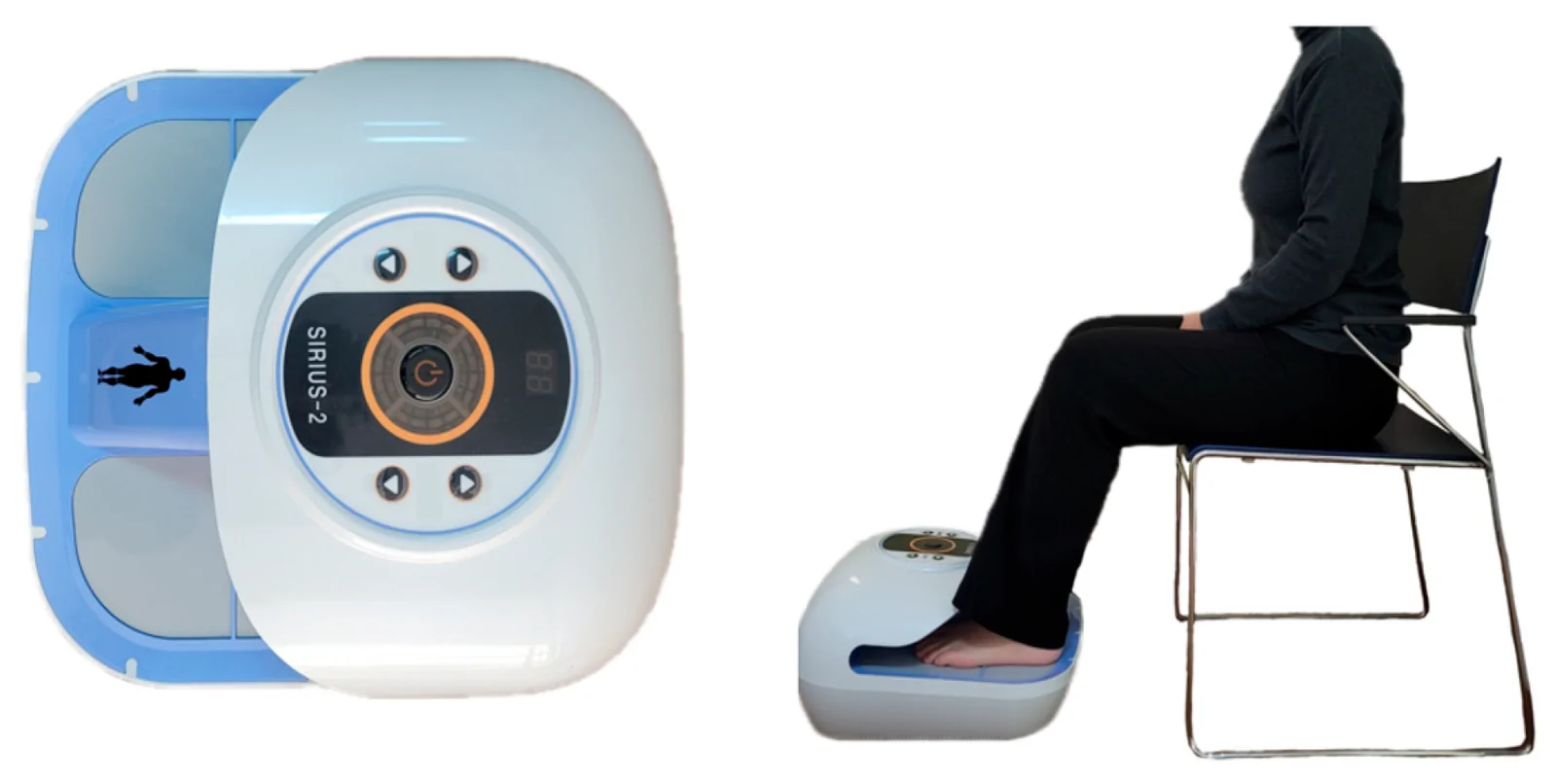
Radiofrequency diathermy device. The device measured 439.06 mm (W) × 400 mm (D) × 135 mm (H) (±10%) and weighed 6.83 kg.

The device used was the SIRIUS RFD system (Class III Medical Device, License No. 19-346; Gemtech Technologies Co., Ltd., Changwon-si, Republic of Korea). The manufacturer calibrated the device on 20 August 2020, approximately 1 month before the intervention began. Participants sat barefoot on a chair with their feet directly placed on the device (Figure 2).

The intervention was administered under standardized conditions according to the manufacturer’s operating procedures. For the active condition, RFD was delivered for 30 min in FOOT mode at intensity level 5, with an output frequency of 470 kHz, a load resistance of 500 Ω, and a voltage of 186.85 Vrms (±10%). The sham RFD device was identical in appearance to the active device and operated at the same frequency (470 kHz), but its output voltage was set to 0.74 Vrms to minimize radiofrequency energy delivery and associated thermal effects. The electrical parameters of the active and sham RFD devices are presented in Table 1.

Because active RFD could produce perceptible thermal sensations, complete participant blinding could not be guaranteed. However, participants were not informed of their treatment sequence, and all outcome assessments were performed by a blinded assessor who was not involved in administering the intervention. The success of participant blinding was not formally assessed.

### 2.5. Assessment and Outcome Measures

During the three sessions under both the active and sham conditions, assessments were conducted immediately before and after each intervention session. Repeated measurements were performed throughout the three-session protocol to evaluate both the immediate and cumulative effects of the interventions. Skin temperature was assessed immediately after treatment, followed by VAS and body weight assessments; circumference and leg volume measurements began approximately 30 min after treatment. Before the second intervention period, participants were re-evaluated to confirm their continued eligibility, including the absence of menstruation and other temporary exclusion conditions. The assessor was a licensed occupational therapist who had received training in the standardized assessment procedures before the initiation of the study. The assessor remained unaware of the treatment allocation throughout data collection and analysis.

Following completion of each intervention session, skin temperature was assessed first using digital infrared thermographic imaging (DITI). A DITI system (T-1000 HD; MESH Co., Ltd., Seoul, Republic of Korea) was used to measure skin temperature as an indirect indicator of deep tissue heating and peripheral circulation following RFD application. Temperature measurements were obtained from predefined regions of interest (ROIs), including the bilateral anterior thighs, anterior shins, posterior thighs, posterior calves, dorsal feet, and plantar feet, yielding a total of 12 ROIs. To minimize the influence of environmental factors on skin temperature, all assessments were performed under standardized conditions, with room temperature maintained at 20.0–23.0 °C and relative humidity at 30–75% [38]. DITI has demonstrated acceptable reproducibility in previous studies [38], and standardized lower-extremity DITI protocols have shown reasonable measurement consistency under controlled examination conditions [39]. To enhance reproducibility, the same trained assessor performed all assessments using standardized procedures, predefined anatomical landmarks or ROIs, and consistent environmental conditions.

Following DITI assessment, perceived lower extremity pain was evaluated using a visual analog scale (VAS), and body weight was measured. Subjective pain related to edema was evaluated using a 10-point VAS, with higher scores indicating greater perceived discomfort [40]. Body weight was measured using a calibrated digital scale (InBody 270; InBody Co., Ltd., Seoul, Republic of Korea) and was included as an additional physiological outcome.

Lower extremity circumference was measured at three anatomical landmarks: the lower calf, 7 cm proximal to the midpoint of the medial malleolus; the region behind the medial malleolus; and the dorsum of the foot [41,42]. A tension-controlled measuring tape (Gulick II, Lafayette Instrument Company, Lafayette, IN, USA) was used to reduce measurement variability associated with differences in tape tension. All measurements were performed with participants seated comfortably in a chair, with both feet resting on the floor and the hips, knees, and ankles maintained at approximately 90° of flexion.

Leg volume was measured using a volumeter (Patterson Medical, Warrenville, IL, USA). The device consists of a rectangular acrylic tank (35 × 18 × 31 cm; W × D × H) with a cylindrical overflow spout located 25 cm above the base. Before each measurement, the tank was filled with water to the level of the overflow spout, corresponding to a water depth of 25 cm. Each lower extremity was slowly immersed in the tank to a standardized depth. The displaced water was collected in a graduated cylinder and its volume measured in milliliters (mL) [41]. Previous studies have demonstrated acceptable reliability of water displacement volumetry and standardized circumference measurements for quantifying peripheral edema [41,42].

All assessments were conducted at a consistent time of day for each participant and under standardized environmental conditions to reduce measurement variability. All outcome measurements were performed by the same blinded assessor using a standardized measurement protocol.

Adverse events, including potential RFD-related events such as low-temperature burns, dermatitis, dizziness, and fever, were monitored throughout the study using participant-reported symptoms and investigator observations.

### 2.6. Statistical Analysis

All statistical analyses were conducted using IBM SPSS Statistics (version 23.0; IBM Corp., Armonk, NY, USA) and Python (version 3.10.12; Python Software Foundation, Wilmington, DE, USA). The primary outcome was the change in leg volume measured by water displacement. Secondary outcomes included pain intensity measured using the VAS, body weight, circumference measurements, and skin temperature.

Because of the exploratory nature of this pilot study and the relatively small sample size, nonparametric statistical methods were selected for the primary between-condition analyses. Demographic characteristics were compared between the Active–Sham and Sham–Active sequence groups using the Mann–Whitney U test. Baseline comparability between the two treatment conditions was assessed using the Wilcoxon signed-rank test for paired samples. For descriptive within-condition pre–post comparisons, values were averaged across the three sessions, and the Wilcoxon signed-rank test was used.

For between-condition comparisons, pre–post changes averaged across the three sessions were analyzed using the Quade test adjusted for baseline values. Effect sizes for between-condition differences were estimated as standardized mean differences (Cohen’s d) calculated using the pooled standard deviation of the averaged pre–post changes.

To account for the crossover design and repeated measurements, linear mixed models (LMMs) were fitted with participants included as a random effect. Treatment condition, time, period, and sequence were included as fixed effects. Period and sequence effects were examined to assess potential period- and order-related influences associated with the crossover design. As a post hoc assessment of potential carryover effects, subject-level pre–post changes were first averaged across the three intervention sessions within each treatment condition. The summed treatment responses across the two crossover periods were then compared between the Active–Sham and Sham–Active sequences using the Mann–Whitney U test. The interaction between treatment condition and time was used to assess differences in patterns of change over time between the active and sham conditions.

All randomized participants who completed both treatment conditions were included in the analyses. No outcome data were missing; therefore, no imputation was required. The primary outcome was analyzed without adjustment for multiple comparisons. Secondary outcomes were adjusted using the Benjamini–Hochberg false discovery rate procedure. All tests were two-sided, and statistical significance was set at *p* < 0.05.

The authors used ChatGPT-5.5 (OpenAI) for language editing and grammatical revision of the manuscript. All scientific content, data analysis, interpretations, and conclusions were developed and verified by the authors.

## 3. Results

### 3.1. Participant Characteristics

Fifteen participants were enrolled in the study. One participant withdrew for personal reasons, resulting in 14 participants who completed the study and were included in the final analysis. Owing to the crossover design, each participant served as her own control and contributed data under both the active and sham conditions. The 14 participants had a mean age of 53.43 ± 10.55 years and an average daily standing time of 7.64 ± 1.65 h.

Baseline measurements are presented by treatment condition in Table 2, with additional comparisons of foot and ankle circumference and regional skin temperature provided in Supplementary Table S1, Panel A. Baseline values at the beginning of each treatment condition did not differ significantly between the active and sham conditions. Sequence-specific demographic characteristics and selected baseline outcome measures are presented separately in Supplementary Table S1, Panel B. In addition, no significant period or sequence effects were observed (Supplementary Table S2, Panel A). Post hoc analyses did not detect statistically significant carryover effects for the primary or selected secondary outcomes (Supplementary Table S2, Panel B).

### 3.2. Within-Condition Changes

Table 3 summarizes within-condition pre- and postintervention comparisons of the principal outcome measures, averaged across the three sessions, under the active and sham conditions. Detailed results for regional skin temperature are provided in Supplementary Table S2.

#### 3.2.1. Active Condition

Descriptive within-condition analyses showed significant pre–post changes under the active condition. For the primary outcome, leg volume decreased bilaterally following the intervention. The mean right leg volume decreased from 1737.76 ± 77.32 mL to 1633.76 ± 112.08 mL (Z = −3.30, *p* < 0.001), whereas the mean left leg volume decreased from 1726.96 ± 72.81 mL to 1645.81 ± 53.94 mL (Z = −3.04, *p* < 0.001).

**Figure 3:**
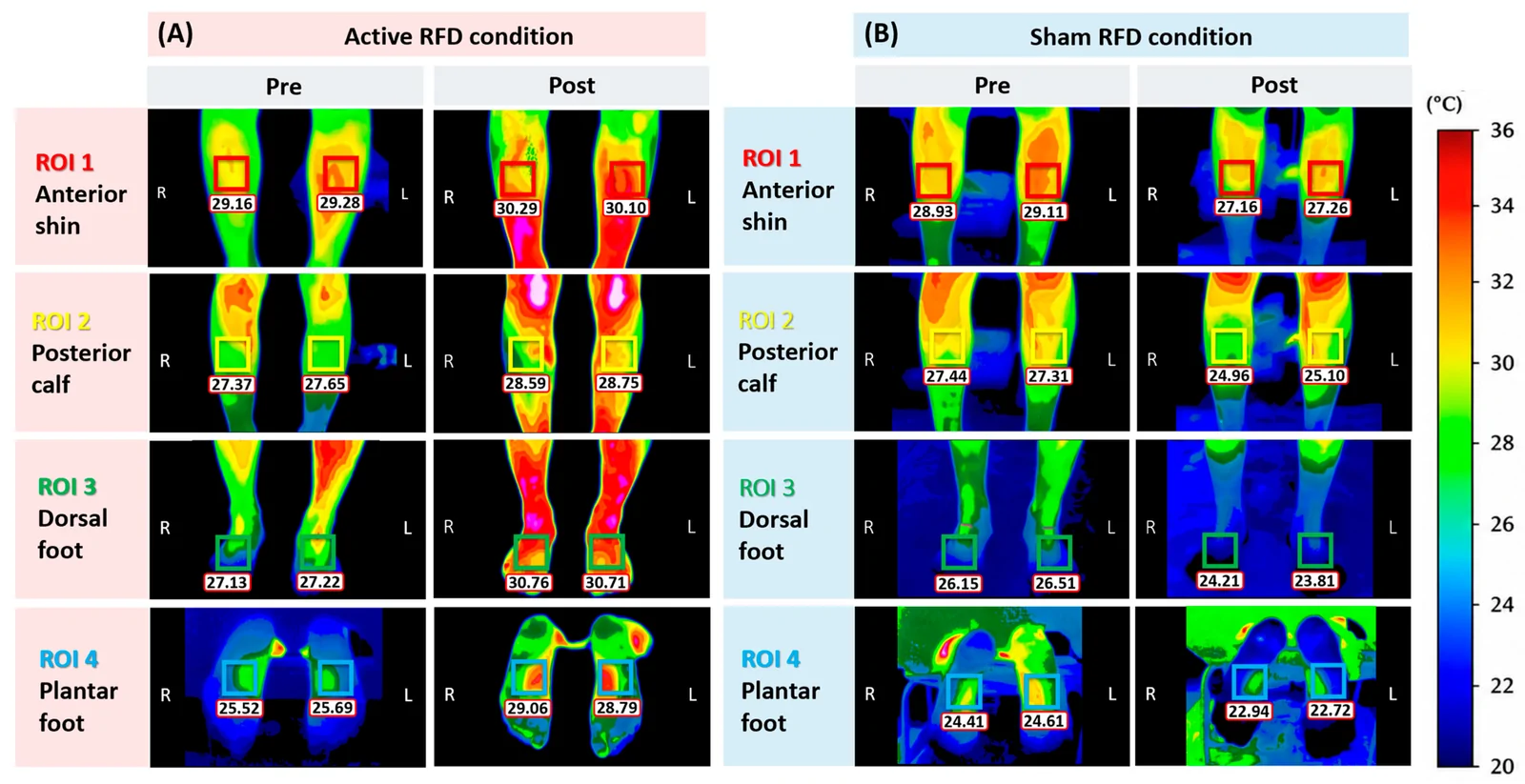
Representative thermographic images before and after the intervention under the active RFD (**A**) and sham (**B**) conditions. Colored boxes indicate predefined regions of interest (ROIs) used to assess skin temperature. The anterior shin ROI was positioned at the midpoint between the tibial tuberosity and the medial malleolus; the posterior calf ROI, at the midpoint between the popliteal crease and the heel; the dorsal foot ROI, over the central midfoot between the second and third metatarsals; and the plantar foot ROI, at the central plantar surface between the heel and the metatarsal heads. A circular ROI approximately 2 cm in diameter was applied consistently at each anatomical location before and after the intervention. Numerical values indicate skin temperature (°C). RFD, radiofrequency diathermy; R, right lower limb; L, left lower limb.

Among the secondary outcomes, significant reductions were observed in pain intensity, body weight, and bilateral calf circumference (all *p* < 0.05). In addition, skin temperature increased significantly in several regions, including the right foot, left foot, and left posterior calf (*p* < 0.05). Representative thermographic images obtained before and immediately after the intervention under the active and sham conditions are shown in Figure 3.

#### 3.2.2. Sham Condition

In contrast, minimal within-condition changes were observed under the sham condition. No significant changes in leg volume were observed. Likewise, pain intensity, body weight, and circumference measurements showed no significant changes. However, skin temperature decreased significantly in several regions, including the anterior shins, posterior calves, and dorsal feet (all *p* < 0.05).

### 3.3. Between-Condition Comparisons of Pre–Post Changes

Baseline-adjusted between-condition comparisons used pre–post changes averaged across the three intervention sessions. For the primary outcome, leg volume decreased more under the active than under the sham condition in both legs. Mean right leg volume changes were −104.0 ± 80.05 mL under the active condition and −14.53 ± 62.87 mL under the sham condition (*p* = 0.013). Corresponding left leg volume changes were −81.14 ± 51.88 mL and 17.37 ± 42.81 mL, respectively (*p* < 0.001).

Among the secondary outcomes, reductions in pain intensity, body weight, and right calf circumference were greater under the active condition after adjustment for multiple comparisons (*p* < 0.05). Skin temperature increases were also significantly greater under the active than under the sham condition in all measured regions except the bilateral anterior thighs (all *p* < 0.05). Detailed between-condition comparisons of the main outcomes are presented in Table 4; additional results for foot and ankle circumference and regional skin temperature are provided in Supplementary Table S3. Regional skin temperature changes are summarized visually in Figure S1.

### 3.4. Linear Mixed Model Analysis of Condition × Time Interactions

Linear mixed models were used to examine whether changes from preintervention to postintervention differed between the active and sham conditions. For the primary outcome, significant condition × time interaction effects were identified for leg volume in both the right leg (*p* < 0.001) and the left leg (*p* = 0.014). Among the secondary outcomes, no significant interaction effects were observed for body weight, calf circumference, or VAS (*p* = 0.054 for VAS). Significant interaction effects for regional skin temperature were observed in most measured regions except the anterior thighs (all *p* < 0.05). Detailed results are presented in Supplementary Table S4, and the main linear mixed model results are summarized in Table 5.

### 3.5. Adverse Events

No adverse events were reported during the treatment period.

## 4. Discussion

The present study explored short-term changes in leg volume and related physiological measures following RFD in women with idiopathic lower extremity edema. Compared with the sham condition, the active condition was associated with greater reductions in bilateral leg volume, pain intensity, body weight, and selected circumference measurements. Skin temperature generally increased under the active condition and decreased under the sham condition. Linear mixed model analyses also showed significant condition × time interactions for leg volume. These findings suggest that RFD may induce short-term physiological responses relevant to idiopathic lower extremity edema.

The primary finding was a reduction in bilateral leg volume under the active condition. At frequencies below 1 MHz, including the 470 kHz used here, RFD induces ionic movement within biological tissues. The resulting resistive energy dissipation converts electromagnetic energy into heat, increasing tissue temperature [13,19]. Studies of 448 kHz radiofrequency therapy have reported improved regional circulation, with increases in skin temperature, skin blood flow, and microvascular perfusion following treatment [43]. These thermally induced changes in regional blood flow and microcirculation may facilitate venous outflow from the distal lower extremities [44]. Improved venous outflow has been associated with reductions in lower extremity swelling in previous clinical studies [45,46]. Previous studies have also shown that lymphatic contraction frequency and lymph flow vary with temperature [47], and a systematic review of human lymphatic physiology reported that heat exposure may increase lymphatic contraction frequency [48]. Enhanced lymphatic contractile activity may facilitate lymph transport and interstitial fluid clearance, potentially reducing tissue fluid accumulation and edema [47,49]. The reductions in leg volume and increases in skin temperature observed after active RFD are consistent with these findings.

Similar findings have been reported for noninvasive radiofrequency-based therapies, including capacitive and resistive energy transfer (TECAR). TECAR therapy was associated with reduced lower extremity volume in patients with lower extremity lymphedema and with greater reductions in lower extremity circumference in women with lipedema when added to standard conservative care [30,31]. These findings are consistent with the short-term leg volume reductions associated with active RFD in this study. Given the lack of previous studies specifically examining RFD in patients with idiopathic edema, however, the present findings should be interpreted as preliminary evidence that RFD-induced thermal responses may influence fluid regulation in this population.

A recent systematic review and meta-analysis of noninvasive radiofrequency therapy for lower extremity conditions reported preliminary evidence of limb volume reduction in patients with lipedema and lymphedema [27]. An earlier meta-analysis of pulsed radiofrequency energy treatment also found significant overall evidence of edema reduction in postoperative and non-postoperative clinical conditions [28]. The preceding reviews suggested that such effects may be related to thermal increases in local perfusion and to non-thermal influences on inflammatory exudate clearance and lymphatic drainage; however, these mechanisms were not directly evaluated in the included trials and therefore remain unconfirmed.

Changes in the secondary outcomes of body weight and circumference were generally consistent in direction with those in leg volume. The reductions in lower extremity circumference under the active condition are consistent with decreases reported in previous studies of radiofrequency-based therapies for edema-related conditions [31]. However, this study cannot determine whether the change in body weight reflected systemic fluid shifts, short-term changes in hydration, or normal physiological variation. Body weight and circumference outcomes should therefore be interpreted as supportive findings alongside the primary outcome of leg volume.

Pain intensity, a secondary outcome, also decreased under the active condition. Previous studies have reported that radiofrequency-based interventions may reduce pain [14,21,25,50]. The analgesic effects of radiofrequency-based therapy have been attributed primarily to thermally mediated physiological responses. Increased tissue temperature may promote local vasodilation and blood flow, facilitate metabolic exchange, reduce muscle spasm and soft-tissue tension, and increase tissue extensibility, thereby potentially reducing nociceptive input and pain [21]. These physiological effects of RFD may also explain the reduction in pain intensity observed under the active condition. In addition, pain associated with idiopathic lower extremity edema may arise from increased local tissue pressure, soft tissue distension, and circulatory congestion secondary to fluid accumulation [1]. Thus, the concurrent reduction in leg volume may also have contributed to lower pain intensity by reducing local tissue pressure and distension. However, VAS scores are self-reported and may be influenced by expectancy effects or incomplete participant blinding. Moreover, the condition × time interaction for VAS-measured pain intensity did not reach conventional statistical significance. The pain-related findings should therefore be interpreted as exploratory.

This study evaluated the short-term effects of RFD administered once daily for 3 consecutive days. The effects of radiofrequency therapy may depend on treatment timing and repetition. Acute physiological studies have shown that a single application of 448 kHz capacitive–resistive monopolar radiofrequency can increase skin temperature and skin blood flow, with thermal effects persisting for at least 20–45 min after treatment [13,37]. In contrast, clinical studies of repeated treatment have generally involved multiple sessions over several weeks. In patients with lower-limb lymphedema, six TECAR sessions produced significant reductions in whole-limb and thigh volume [30]. Similarly, in women with stage 2 lipedema, six TECAR sessions over 3 weeks reduced lower-limb circumference and improved pain in the short term. However, at 3 months, sustained improvements were limited to selected circumference measurements, and the analgesic effect was no longer significant [31]. Evidence on long-term maintenance therapy remains limited, and no specific monthly treatment frequency has been established for sustaining the effects of radiofrequency therapy. Therefore, the present 3-day findings should be interpreted as short-term effects; further studies are needed to determine the optimal treatment interval and maintenance frequency for sustaining longer-term benefits, particularly in patients with idiopathic lower extremity edema.

The potential clinical relevance of these findings should also be considered. Although the changes observed in the present study were short-term, reductions in leg volume and pain following RFD may be meaningful for patients who experience recurrent or chronic edema-related symptoms. If future studies confirm its efficacy, RFD may represent a supportive management option for idiopathic lower extremity edema because of its noninvasive application, standardized delivery, and favorable safety profile observed in this study. Its clinical relevance may thus lie in its potential as a simple, repeatable, noninvasive adjunct to existing symptom-management strategies for recurrent lower extremity edema.

No adverse events were reported during the intervention period, suggesting that short-term RFD application was well tolerated in this small pilot study conducted under strict eligibility criteria. However, the sample size was limited, and individuals with potential risk factors, such as cardiovascular disease, pregnancy, active bleeding, or uncontrolled hypertension, were excluded. Therefore, the safety of RFD cannot be generalized to broader clinical populations based on the present findings alone. Future studies should evaluate safety, tolerability, and clinical effectiveness in larger and more diverse populations, particularly with repeated or longer-term application.

Some limitations should be acknowledged. First, the small sample size and short intervention period limited statistical power and generalizability. Second, although no significant period, sequence, or carryover effects were detected, the small sample size limited the statistical power of these assessments; therefore, residual carryover effects cannot be completely excluded. Period and sequence effects were examined, but the possibility of residual effects remains. Third, complete participant blinding was difficult because active RFD produced perceptible thermal sensations, and blinding success was not formally assessed.

Despite these limitations, the present study provides preliminary evidence regarding short-term physiological responses to RFD in women with idiopathic lower extremity edema. Future studies with larger samples, longer follow-up periods, and direct physiological measurements of blood flow and lymphatic function are needed to clarify the mechanisms underlying the observed volume changes and to determine the clinical relevance of RFD for idiopathic lower extremity edema.

## 5. Conclusions

In women with idiopathic lower extremity edema, the active condition was associated with greater short-term reductions in bilateral leg volume than the sham condition. Changes in skin temperature and selected physiological measures were consistent with short-term physiological responses to RFD in this population. Further studies with larger samples, longer follow-up periods, and direct physiological assessments are needed to confirm these findings and clarify their clinical relevance.

## Figures and Tables

**Table 1:** Electrical parameters of radiofrequency diathermy device.

Parameter	Active Condition	Sham Condition
Mode	FOOT	FOOT
Output frequency (kHz)	470	470
Intensity level	5	sham
Load resistance (Ω)	500	500
Voltage (Vrms)	186.85 (±10%)	0.74 (±10%)
Treatment duration (min)	30	30

**Table 2:** Baseline outcome measurements by treatment condition (*N* = 14).

Variable	Active Condition	Sham Condition	*p*-Value
Right leg volume (mL)	1778.72 ± 60.48	1743.41 ± 56.55	0.101
Left leg volume (mL)	1775.44 ± 86.15	1703.99 ± 91.73	0.058
VAS score	3.29 ± 0.73	3.07 ± 0.73	0.499
Body weight (kg)	62.29 ± 7.07	61.74 ± 7.00	0.463
Right calf circumference (cm)	27.19 ± 3.71	26.14 ± 3.17	0.090
Left calf circumference (cm)	27.18 ± 3.70	26.54 ± 2.90	0.135

Note: Values are presented as mean ± SD unless otherwise indicated. VAS, visual analog scale; SD, standard deviation.

**Table 3:** Within-condition pre–post comparisons of outcome measures averaged across the three intervention sessions.

Variable	Pre	Post	Z Value	*p* Value
Active condition				
Right leg volume (mL)	1737.76 ± 77.32	1633.76 ± 112.08	−3.30	**<0.001**
Left leg volume (mL)	1726.96 ± 72.81	1645.81 ± 53.94	−3.04	**<0.001**
VAS score	3.95 ± 0.50	2.38 ± 0.64	−2.94	**0.002**
Body weight (kg)	62.08 ± 7.23	61.80 ± 7.23	−2.54	**0.009**
Right calf circumference (cm)	26.91 ± 3.31	26.32 ± 3.38	−2.04	**0.042**
Left calf circumference (cm)	26.89 ± 3.33	26.28 ± 3.29	−2.73	**0.004**
Sham condition				
Right leg volume (mL)	1715.35 ± 78.34	1700.83 ± 64.89	−0.85	0.426
Left leg volume (mL)	1711.32 ± 55.51	1728.69 ± 76.21	−0.72	0.502
VAS score	3.02 ± 0.31	2.88 ± 0.45	−1.54	0.148
Body weight (kg)	61.94 ± 6.68	61.98 ± 6.63	−0.91	0.391
Right calf circumference (cm)	26.43 ± 2.96	26.41 ± 2.99	−0.81	0.471
Left calf circumference (cm)	26.36 ± 3.10	26.34 ± 3.03	−0.28	0.808

Note: Values are presented as mean ± SD unless otherwise indicated. Bold values indicate statistical significance at *p* < 0.05. VAS, visual analog scale; SD, standard deviation.

**Table 4:** Between-condition comparisons of pre–post changes averaged across the three intervention sessions.

Outcome	Active Condition	Sham Condition	*p* Value	Adjusted *p* Value	Cohen’s d
Right leg volume (mL)	−104.0 ± 80.05	−14.53 ± 62.87	**0.013**	—	−1.45
Left leg volume (mL)	−81.14 ± 51.88	17.37 ± 42.81	**<0.001**	—	−1.52
VAS score	−1.57 ± 0.48	−0.14 ± 0.31	**0.022**	**0.033**	−2.10
Body weight (kg)	−0.28 ± 0.34	0.04 ± 0.24	**0.017**	**0.027**	−0.92
Right calf circumference (cm)	−0.59 ± 0.67	−0.02 ± 0.25	**0.030**	**0.036**	−0.98
Left calf circumference (cm)	−0.61 ± 0.69	−0.02 ± 0.45	**0.049**	0.057	−0.95

Note: Values are the mean change ± SD, calculated from participant-level pre–post changes averaged across the three sessions. Negative values indicate reductions. *p* values are from paired between-condition comparisons of the averaged pre–post changes. Adjusted *p* values for secondary outcomes were calculated using the Benjamini–Hochberg procedure. Cohen’s d was calculated using the pooled standard deviation of the averaged pre–post changes. Bold values indicate statistical significance at *p* < 0.05. SD, standard deviation; VAS, visual analog scale.

**Table 5:** Linear mixed model analysis of condition × time interaction effects for primary and selected secondary outcomes.

Outcome	Coefficient (Condition × Time)	*p* Value
Right leg volume (mL)	−100.876	**<0.001**
Left leg volume (mL)	−64.977	**0.014**
VAS score	−0.429	0.054
Body weight (kg)	−0.321	0.440
Right calf circumference (cm)	−0.266	0.448
Left calf circumference (cm)	−0.468	0.224

Note: Coefficients represent condition × time interaction effects estimated using linear mixed models. Bold values indicate statistical significance at *p* < 0.05. VAS, visual analog scale.

## Data Availability

The data presented in this study are available from the corresponding author upon reasonable request.

## Supplementary Materials

The following supporting information can be downloaded at: https://www.mdpi.com/article/10.3390/jcm15207974/s1, Table S1: Baseline characteristics and outcome measures by treatment sequence and treatment condition; Table S2: Analysis of period, sequence, and potential carryover effects in the crossover design; Table S3: Within-condition pre–post comparisons of additional outcomes averaged across three sessions; Table S4: Between-condition comparisons of pre–post changes averaged across the three intervention sessions; Table S5: Linear mixed model analysis of condition × time interaction effects for additional outcomes; Figure S1: Heat map of mean skin temperature changes (postintervention minus preintervention) across predefined anatomical regions under the active and sham RFD conditions. Positive values indicate increases in skin temperature; negative values indicate decreases. Values are mean changes averaged across the three intervention sessions. RFD, radiofrequency diathermy.

## Abbreviations

The following abbreviations are used in this manuscript:

RFD	Radiofrequency diathermy
DITI	Digital infrared thermographic imaging
ROI	Region of interest
VAS	Visual analog scale
LMM	Linear mixed model
TECAR	Capacitive and resistive energy transfer
