# Supplementary material for: Short-Term Changes in Lower Extremity Volume After Radiofrequency Diathermy in Women with Idiopathic Lower Extremity Edema: A Randomized Crossover Trial
Source: J Clin Med. 2026 Oct 15;15(20):7974. doi: 10.3390/jcm15207974 (PMC13706332; doi:10.3390/jcm15207974)
Supplement: Supplementary file 1 [file jcm-15-07974-s001.zip › Table_S1_260930.pdf]

Supplementary Table S1. Baseline characteristics and outcome measures by treatment sequence and treatment condition

Panel A. Baseline comparisons of foot and ankle circumference and regional skin temperature between the active and sham conditions

- **Foot and ankle circumference (cm)**

Outcome	Active condition	Sham condition	*p* value
Right foot circumference	21.84 ± 1.10	21.31 ± 1.19	0.053
Left foot circumference	21.43 ± 2.33	21.14 ± 1.35	0.855
Right ankle circumference	21.91 ± 1.35	21.35 ± 1.82	0.245
Left ankle circumference	21.68 ± 2.01	21.56 ± 1.94	0.855

- **Regional skin temperature (°C)**

Outcome	Active condition	Sham condition	*p* value
Left anterior thigh temperature	27.97 ± 1.37	27.35 ± 2.31	0.670
Right anterior thigh temperature	27.94 ± 1.47	26.87 ± 2.12	0.217
Left anterior shin temperature	29.14 ± 1.30	28.94 ± 1.53	0.626
Right anterior shin temperature	29.27 ± 1.66	29.11 ± 2.06	0.715
Left posterior thigh temperature	27.55 ± 1.20	27.62 ± 1.39	0.542
Right posterior thigh temperature	28.06 ± 0.95	28.07 ± 1.49	0.583
Left posterior calf temperature	27.38 ± 1.49	27.39 ± 2.23	0.855
Right posterior calf temperature	27.72 ± 1.95	27.41 ± 1.98	0.463
Left dorsal foot temperature	27.29 ± 3.64	26.68 ± 3.12	0.326
Right dorsal foot temperature	26.91 ± 3.62	26.14 ± 3.74	0.217
Left plantar foot temperature	25.54 ± 2.97	24.82 ± 4.59	0.463
Right plantar foot temperature	25.71 ± 2.94	24.65 ± 3.52	0.391

NOTE: Values are presented as mean change ± SD, calculated from subject-level averaged pre–post changes across three sessions. Positive values indicate increases and negative values indicate decreases. *p* values represent paired between-condition comparisons

Panel B. Demographic characteristics and selected baseline outcome measures by randomized treatment sequence

**Characteristic**	**Active–Sham sequence (n = 7)**	**Sham–Active sequence (n = 7)**	**U**	***p* value**
Age (years)	50.71 ± 13.26	56.14 ± 6.94	22	0.798
Average daily standing time (h)	6.57 ± 0.98	8.71 ± 1.50	5.5	**0.015**
Right leg volume (mL)	1779.41 ± 56.29	1741.88 ± 90.94	30	0.535
Left leg volume (mL)	1774.83 ± 107.45	1705.36 ± 80.45	36	0.165
VAS score	3.57 ± 0.53	3.29 ± 0.76	29.5	0.52
Body weight (kg)	61.88 ± 6.13	61.50 ± 8.31	25	1
Right calf circumference (cm)	26.61 ± 4.49	25.25 ± 2.11	25	1
Left calf circumference (cm)	26.88 ± 4.40	25.49 ± 1.83	26	0.902

NOTE: Values are presented as mean ± SD. Demographic characteristics and selected baseline outcome measures were compared between the Active–Sham and Sham–Active sequence groups using the Mann–Whitney U test. Baseline outcome measures represent preintervention values obtained at the beginning of the first treatment period. Bold values indicate statistical significance at $p < 0.05$. VAS, visual analog scale; SD, standard deviation.
