# Supplementary material for: Short-Term Changes in Lower Extremity Volume After Radiofrequency Diathermy in Women with Idiopathic Lower Extremity Edema: A Randomized Crossover Trial
Source: J Clin Med. 2026 Oct 15;15(20):7974. doi: 10.3390/jcm15207974 (PMC13706332; doi:10.3390/jcm15207974)
Supplement: Supplementary file 1 [file jcm-15-07974-s001.zip › Table_S2_260930.pdf]

Supplementary Table S2. Analysis of period, sequence, and potential carryover effects in the crossover design

Panel A. Period and sequence effects from linear mixed models

Outcome	Period effect coefficient	*p* value	Sequence effect coefficient	*p* value
Right leg volume (mL)	-4.472	0.682	-21.672	0.468
Left leg volume (mL)	5.045	0.709	-34.577	0.336
VAS score	-0.119	0.284	-0.071	0.709
Body weight (kg)	0.123	0.545	0.138	0.971
Right calf circumference (cm)	0.115	0.587	-0.273	0.875
Left calf circumference (cm)	0.204	0.249	-0.225	0.895

NOTE: Coefficients and p values were estimated using linear mixed models with participant as a random effect and treatment condition, time, period, sequence, and condition × time as fixed effects. Time indicated whether the measurement was obtained before or after the intervention. Period indicated whether the measurement was obtained during the first or second treatment period of the crossover trial, and sequence indicated the order of treatment administration (Active–Sham or Sham–Active). Period coefficient represents Period 2 relative to Period 1; sequence coefficient represents Sequence 2 relative to Sequence 1.

Panel B. Post hoc assessment of potential carryover effects for primary and selected secondary outcomes

Outcome	Active–Sham sequence (n = 7), Mean ± SD	Sham–Active sequence (n = 7), Mean ± SD	Carryover test *p*
Right leg volume (mL)	−150.54 ± 97.86	−91.69 ± 40.71	0.318
Left leg volume (mL)	−109.99 ± 72.80	−42.86 ± 53.16	0.097
VAS	−0.81 ± 0.69	−0.62 ± 0.36	0.383
Body weight (kg)	−0.19 ± 0.34	−0.29 ± 0.46	1.000
Right calf circumference (cm)	−0.56 ± 0.83	−0.22 ± 0.23	0.535
Left calf circumference (cm)	−0.46 ± 1.19	−0.04 ± 0.62	0.456

NOTE: Values are presented as mean ± SD. Potential carryover effects were assessed post hoc by comparing summed treatment responses across the two crossover periods between the Active–Sham and Sham–Active sequences using the Mann–Whitney U test. Treatment responses were calculated from subject-level pre–post changes averaged across the three intervention sessions.
