# Supplementary material for: Short-Term Changes in Lower Extremity Volume After Radiofrequency Diathermy in Women with Idiopathic Lower Extremity Edema: A Randomized Crossover Trial
Source: J Clin Med. 2026 Oct 15;15(20):7974. doi: 10.3390/jcm15207974 (PMC13706332; doi:10.3390/jcm15207974)
Supplement: Supplementary file 1 [file jcm-15-07974-s001.zip › Table_S3_260930.pdf]

Supplementary Table S3. Within-condition pre–post comparisons of additional outcomes averaged across three sessions

Panel A. Foot and ankle circumference (cm)

Variable	Pre (mean ± SD)	Post (mean ± SD)	*p* value
**Active condition**			
Right foot circumference	21.71 ± 1.22	21.18 ± 1.18	0.268
Left foot circumference	21.70 ± 1.22	21.35 ± 1.17	0.502
Right ankle circumference	21.45 ± 2.07	21.14 ± 2.22	0.626
Left ankle circumference	21.38 ± 2.08	21.16 ± 2.12	0.104
**Sham condition**			
Right foot circumference	21.27 ± 1.08	21.25 ± 1.19	0.808
Left foot circumference	21.25 ± 1.25	21.37 ± 1.38	0.296
Right ankle circumference	21.21 ± 1.58	21.18 ± 1.63	1.000
Left ankle circumference	21.34 ± 1.81	21.37 ± 1.61	0.903

Panel B. Regional skin temperature (°C)

Variable	Pre (mean ± SD)	Post (mean ± SD)	*p* value
**Active condition**			
Right anterior thigh temperature	27.80 ± 1.31	28.29 ± 1.29	0.133
Left anterior thigh temperature	27.79 ± 1.23	28.22 ± 1.37	0.109
Right anterior shin temperature	29.16 ± 1.20	30.29 ± 1.48	0.016
Left anterior shin temperature	29.28 ± 1.30	30.10 ± 1.70	0.068
Right posterior thigh temperature	28.01 ± 0.91	28.60 ± 1.58	0.158
Left posterior thigh temperature	27.72 ± 1.07	28.26 ± 1.56	0.124
Right posterior calf temperature	27.65 ± 1.22	28.75 ± 2.24	0.069

Variable	Pre (mean ± SD)	Post (mean ± SD)	*p* value
Left posterior calf temperature	27.37 ± 1.00	28.59 ± 1.91	0.025
Right dorsal foot temperature	27.13 ± 3.56	30.76 ± 1.39	<0.001
Left dorsal foot temperature	27.22 ± 3.13	30.71 ± 1.46	0.001
Right plantar foot temperature	25.69 ± 2.66	28.79 ± 2.36	0.002
Left plantar foot temperature	25.52 ± 2.67	29.06 ± 1.96	0.002
**Sham condition**			
Right anterior thigh temperature	26.95 ± 1.81	27.61 ± 1.19	0.078
Left anterior thigh temperature	26.88 ± 2.05	27.64 ± 1.38	0.042
Right anterior shin temperature	28.93 ± 1.60	27.16 ± 1.43	<0.001
Left anterior shin temperature	29.11 ± 1.21	27.26 ± 1.42	<0.001
Right posterior thigh temperature	27.78 ± 1.22	27.37 ± 1.13	0.104
Left posterior thigh temperature	27.71 ± 1.52	27.03 ± 1.29	0.041
Right posterior calf temperature	27.31 ± 1.50	25.10 ± 1.33	<0.001
Left posterior calf temperature	27.44 ± 1.56	24.96 ± 1.28	<0.001
Right dorsal foot temperature	26.15 ± 3.67	24.21 ± 4.51	0.039
Left dorsal foot temperature	26.51 ± 3.37	23.81 ± 4.31	0.011
Right plantar foot temperature	24.61 ± 3.63	22.72 ± 4.11	0.103
Left plantar foot temperature	24.41 ± 4.00	22.94 ± 4.34	0.217

Note: Values are presented as mean ± SD averaged across three sessions. *p* values were obtained from within-condition pre–post comparisons using paired non-parametric tests. Abbreviations: SD, standard deviation.
