# Supplementary material for: Short-Term Changes in Lower Extremity Volume After Radiofrequency Diathermy in Women with Idiopathic Lower Extremity Edema: A Randomized Crossover Trial
Source: J Clin Med. 2026 Oct 15;15(20):7974. doi: 10.3390/jcm15207974 (PMC13706332; doi:10.3390/jcm15207974)
Supplement: Supplementary file 1 [file jcm-15-07974-s001.zip › Table_S4_260930.pdf]

**Supplementary Table S4. Between-condition comparisons of pre–post changes averaged across the three intervention sessions**

**Panel A.** Foot and ankle circumference (cm)

| Outcome | Active condition mean change ± SD | Sham condition mean change ± SD | *p* value | Adjusted *p* value |
|---|---|---|---|---|
| Right foot circumference | -0.13 ± 0.33 | -0.02 ± 0.21 | 0.463 | 0.618 |
| Left foot circumference | -0.06 ± 0.29 | 0.12 ± 0.43 | 0.296 | 0.592 |
| Right ankle circumference | -0.12 ± 0.80 | -0.03 ± 0.39 | 0.217 | 0.592 |
| Left ankle circumference | -0.22 ± 0.47 | 0.02 ± 0.54 | 0.670 | 0.670 |

**Panel B.** Regional skin temperature (°C)

| Outcome | Active condition mean change ± SD | Sham condition mean change ± SD | *p* value | Adjusted *p* value |
|---|---|---|---|---|
| Left anterior thigh temperature | 0.44 ± 0.78 | 0.76 ± 1.29 | 0.463 | 0.494 |
| Right anterior thigh temperature | 0.49 ± 0.97 | 0.66 ± 1.06 | 0.761 | 0.761 |

| Outcome | Active condition mean change ± SD | Sham condition mean change ± SD | *p* value | Adjusted *p* value |
|---|---|---|---|---|
| Left anterior shin temperature | 0.83 ± 1.55 | -1.84 ± 1.12 | <0.001 | 0.002 |
| Right anterior shin temperature | 1.13 ± 1.23 | -1.76 ± 1.34 | <0.001 | <0.001 |
| Left posterior thigh temperature | 0.54 ± 1.32 | -0.69 ± 1.06 | 0.011 | 0.019 |
| Right posterior thigh temperature | 0.59 ± 1.59 | -0.41 ± 0.89 | 0.030 | 0.036 |
| Left posterior calf temperature | 1.21 ± 1.68 | -2.48 ± 1.61 | <0.001 | <0.001 |
| Right posterior calf temperature | 1.10 ± 1.96 | -2.21 ± 1.29 | <0.001 | 0.002 |
| Left dorsal foot temperature | 3.49 ± 2.94 | -2.71 ± 3.33 | <0.001 | <0.001 |
| Right dorsal foot temperature | 3.64 ± 3.10 | -1.94 ± 3.03 | <0.001 | <0.001 |
| Left plantar foot temperature | 3.54 ± 2.57 | -1.48 ± 4.29 | 0.005 | 0.010 |

| Outcome | Active condition mean change ± SD | Sham condition mean change ± SD | *p* value | Adjusted *p* value |
|---|---|---|---|---|
| Right plantar foot temperature | 3.09 ± 2.39 | -1.89 ± 4.07 | 0.004 | 0.009 |

NOTE: Values are presented as mean change ± SD, calculated from subject-level averaged pre–post changes across three sessions. Positive values indicate increases and negative values indicate decreases. *p* values represent paired between-condition comparisons. Adjusted *p* values were calculated using the Benjamini–Hochberg procedure.
