# Supplementary material for: Short-Term Changes in Lower Extremity Volume After Radiofrequency Diathermy in Women with Idiopathic Lower Extremity Edema: A Randomized Crossover Trial
Source: J Clin Med. 2026 Oct 15;15(20):7974. doi: 10.3390/jcm15207974 (PMC13706332; doi:10.3390/jcm15207974)
Supplement: Supplementary file 1 [file jcm-15-07974-s001.zip › Table_S5_260930.pdf]

Supplementary Table S5. Linear mixed model analysis of condition × time interaction effects for additional outcomes

Panel A. Foot and ankle circumference (cm)

Outcome	Coefficient (condition × time)	*p* value
Right foot circumference	-0.063	0.782
Left foot circumference	-0.230	0.401
Right ankle circumference	0.025	0.946
Left ankle circumference	0.049	0.903

Panel B. Regional skin temperature (°C)

Outcome	Coefficient (condition × time)	*p* value
Right anterior thigh temperature	-0.164	0.691
Left anterior thigh temperature	-0.329	0.431
Right anterior shin temperature	2.893	**<0.001**
Left anterior shin temperature	2.668	**<0.001**
Right posterior thigh temperature	0.993	**0.012**
Left posterior thigh temperature	1.229	**0.002**
Right posterior calf temperature	3.307	**<0.001**
Left posterior calf temperature	3.693	**<0.001**
Right dorsal foot temperature	5.571	**<0.001**
Left dorsal foot temperature	6.200	**<0.001**
Right plantar foot temperature	4.986	**<0.001**
Left plantar foot temperature	5.014	**<0.001**

NOTE: Coefficients represent condition × time interaction effects estimated from linear mixed models. Bold values indicate statistical significance ($p < 0.05$).
